# New Zealand Health Quality & Safety Commission infection prevention and control programmes: evidence for sustained improvement in infection prevention interventions

**DOI:** 10.1186/2047-2994-4-S1-P58

**Published:** 2015-06-16

**Authors:** S Roberts, D Jowitt

**Affiliations:** 1 Health Quality & Safety Commission, Infection Prevention and Control Programme, Auckland, New Zealand

## Introduction

In 2010 the New Zealand Health Quality & Safety Commission was established. Since 2011 the Commission has sponsored three infection prevention and control programmes; improving hand hygiene compliance, reducing central line associated bacteraemia and surgical site infections.

## Objectives

The aim is to report on the progress to date of these quality improvement programmes.

## Methods

Hand Hygiene New Zealand adopted the WHO ‘5 moments for hand hygiene’ approach using Front-line ownership (FLO) improvement methodology, Target CLAB ZERO used the Institute for Healthcare Improvement collaborative methodology and the National Surgical Site Infection Improvement (SSII) Programme has used an inclusive approach utilising the ‘right tool for the right job’. Each programme has a set of process measure and outcome markers against which performance is measured. These, called the Quality and Safety Markers (QSM), are reported to the Minister of Health. Performance over time is used to support quality improvement.

## Results

There has been has been a sustained improvement in hand hygiene compliance. The current target is 75% with the target moving to 80% by June 2015. Improvement in the outcome marker, healthcare-associated *Staphylococcus aureus* bacteraemia, has not clearly been shown.

The Target CLAB ZERO programme resulted in a reduction in central-line associated bacteraemia events in intensive care units to below 1/1000 central line days which has been sustained for over 12 months. Compliance with the CLAB insertion bundle is >90%.

The SSII programme has focused on hip and knee arthroplasty procedures. There has been a significant improvement in the process measures – choice, dose and timing of antimicrobial prophylaxis and alcohol-based skin antisepsis – but it is too early in the programme to see a reduction in the surgical site infection rate.

## Conclusion

These sector-lead, nationally delivered, infection prevention and control quality improvement programmes have resulted in improved outcomes for patients. Whilst it is early in the improvement cycle it appears that the improvement can be sustained.

To be successful these programmes need good clinical leadership, quality improvement expertise and an engaged and committed healthcare workforce.

## Disclosure of interest

None declared.

